# Thermographic Changes following Short-Term High-Intensity Anaerobic Exercise

**DOI:** 10.3390/life13112175

**Published:** 2023-11-07

**Authors:** Nir Fink, Shai Bogomilsky, Avi Raz, Oshrit Hoffer, Mickey Scheinowitz

**Affiliations:** 1 Sylvan Adams Sports Institute, School of Public Health, Sackler Faculty of Medicine, Tel Aviv University, Tel Aviv 6997801, Israel; 2 Department of Biomedical Engineering, Faculty of Engineering, Tel Aviv University, Tel Aviv 6997801, Israel; 3 School of Electrical Engineering, Afeka Tel Aviv Academic College of Engineering, Tel Aviv 6910717, Israel

**Keywords:** thermographic changes, Wingate anaerobic exercise test, body surface temperature, thermal imaging, anaerobic mechanical power output

## Abstract

Current studies report thermographic changes following aerobic or resistance exercise but not short, vigorous anaerobic exercise. Therefore, we investigated body surface temperature changes using thermal imaging following a short session of anaerobic exercise. We studied three different regions of interest (ROIs): the legs, chest, and forehead. Thermal imaging for each participant was performed before and immediately after completing a Wingate anaerobic test and every minute during a 15 min recovery period. Immediately after the test, the maximum temperature was significantly higher in all ROIs (legs, *p* = 0.0323; chest, *p* = 0.0455; forehead, *p* = 0.0444) compared to pre-test values. During the recovery period, both legs showed a significant and continuous temperature increase (right leg, *p* = 0.0272; left leg, *p* = 0.0382), whereas a non-significant drop was noted in the chest and forehead temperatures. Additionally, participants with a lower anaerobic capacity exhibited a higher delta increase in surface leg temperature than participants with higher anaerobic capacities, with a minimal change in surface leg temperature. This is the first study to demonstrate body surface temperature changes following the Wingate anaerobic test. This temperature increase is attributed to the high anaerobic mechanical power outputs achieved by the leg muscles and the time taken for temperature reduction post-exercise.

## 1. Introduction

Thermal imaging is a rapid, noninvasive, and noncontact imaging technique based on electromagnetic radiation in the infrared range that measures changes in the body surface radiation temperature [1,2]. It has been used in several medical applications, including diagnosis, exercise physiology, and sports medicine [1,2,3,4,5,6]. Thermography enables organ characterization in terms of structure, blood flow, and heat transitions by analyzing the surface temperature in real time [1,2,3,4,5,6]. A recent study from our lab has shown that thermal imaging during graded aerobic exercise stress tests, combined with advanced image processing, is correlated with exercise intensity and pulmonary ventilation [7]. Advanced image processing algorithms are also used to measure other parameters, such as temperature distribution [7,8,9].

The Wingate anaerobic test is a short (30 s), high-intensity, “all out” exercise that assesses anaerobic capacity [10,11]. The test is performed on a stationary bike in a seated position. The test is simple to administer, noninvasive, safe, and intended to measure peak and mean anaerobic mechanical power outputs [10,11].

Several studies have investigated thermal changes associated with aerobic exercises [1]. Other studies have measured body surface thermal changes during resistance exercise and have indicated an increase in temperature immediately after exercise [1,12,13,14,15,16,17,18]. However, to the best of our knowledge, no studies have measured body surface temperature during anaerobic exercise stress tests such as the Wingate test. Undergoing high-intensity “all-out” cycling exercise for 30 s causes blood redistribution into the working muscles. However, due to the high-intensity nature of the muscle activity, there is limited blood supply during the exercise itself, and most of the blood will accumulate in the lower limbs after the exercise has ended [12,19,20,21]. Hussain et al. (1996) examined blood flow distribution to the thigh after the Wingate test using Doppler ultrasound and found increased blood flow to the thigh muscles, which returned to normal after 60 min of recovery. This is why this specific mode of exercise testing is unique and differs from physiological and thermal perspectives.

During high-intensity exercise, cardiac output increased by 5- to 6-fold (up to 25–30 L/min) from its resting value (5 L/min). During the Wingate test, most of the blood will be redirected into the working muscles, and therefore, there is a redistribution of blood away from non-working muscles. Additionally, the contracting muscles vasoconstrict during the 30 s of the test, and therefore, temperature changes will occur only after the test has ended, when the muscles are relaxed and vasodilation allows blood to accumulate in the lower limb.

In the current study, we investigated changes in the body surface temperature of the active thigh muscles as well as non-working muscles of the chest and forehead during and following the Wingate test. We hypothesized that, owing to higher cardiac output, blood would be redistributed from the chest and forehead to the lower limbs immediately after the test, resulting in increased surface temperature of the thigh and decreased surface temperature of the chest and forehead. Therefore, our novel study aimed to measure the surface temperatures of the thigh, chest, and forehead following the Wingate anaerobic test and during the recovery period to develop a better understanding of the blood/surface temperature redistribution associated with this type of short-term, high-intensity anaerobic exercise.

## 2. Materials and Methods

The Ethics Committee of Tel Aviv University granted approval for this study (approval number: 0002102-1). Written informed consent was obtained from all study participants, and all procedures adhered to the appropriate guidelines and regulations. 

This study was conducted at the Sylvan Adams Sports Institute at Tel Aviv University. The sample size was calculated using an alpha of 0.05 and 80% power. Healthy active men and women (n = 24) aged 18–38 years were selected for the test. All participants had 1–20 years of experience in cycling, running, or weightlifting. Laboratory environment conditions were kept constant at 21.1 ± 0.6 °C with humidity of 62.7 ± 2.1%, regardless of ambient weather. The participants were acclimated to the environmental conditions for 15 min before the start of the test. A SECA medical Body Composition Analyzer (mBCA) 515 (SECA GmbH & Co. KG, Hamburg, Germany) was used to analyze body composition (body mass, lean body mass, fat mass, and muscle mass). The participants underwent the Wingate anaerobic test in the sitting position on an SRM stationary ergometer (Schoberer Rad Messtechnik, SRM GmbH, Jülich, Germany) adjusted to the height of each participant. The participants performed a 4 min warm-up at 50 watts of continuous pedaling with two sprints of a few seconds at 1 and 3 min. After the warm-up, the participants rested on the SRM bike for one minute before the test began. The participants were instructed to produce the maximum power output they could while maintaining 80 revolutions per minute (RPM) during the 30 s test. After the test, the participants remained seated with no pedaling for 15 min of recovery.

**Figure 1:**
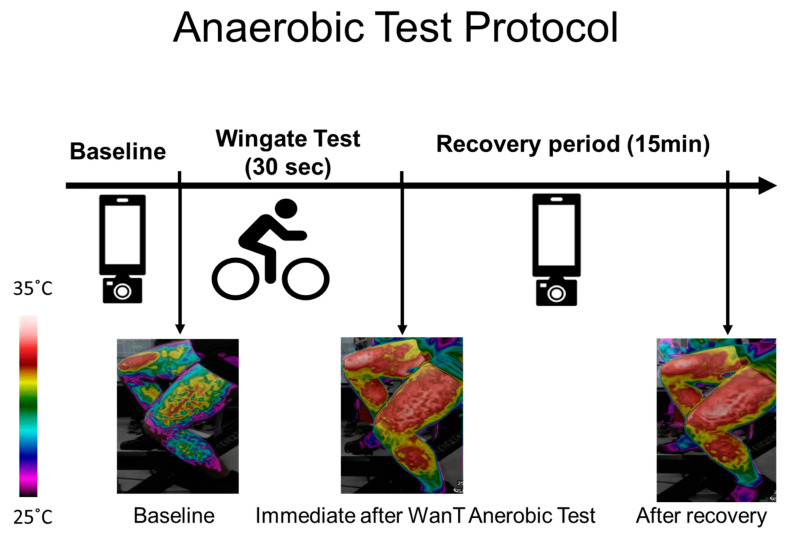
Test procedure and example of an image obtained from the thighs over the course of the experiment. Thermal images were taken at baseline (before the Wingate test - WanT), immediately after the test, and every 60 s during the 15 min recovery period. The increase in surface temperature can be seen via the change in the color bar on the left.

Thermal images of the upper (the chest and forehead in one image) and lower body (both thighs in one image) were captured at rest, before the test (after the warm-up), immediately after the test, and every 60 s during the 15 min recovery period. In each case, a picture of the legs, chest, and forehead was taken (Figure 1).

### 2.1. Thermal Imaging

Thermal images were acquired using a FLIR ONE thermal camera (Teledyne FLIR LLC., Wilsonville, OR, USA). The FLIR ONE thermal camera connects directly to smartphones and offers the following functions: a frame rate frequency of 8.7 Hz, an object temperature range of −20 °C to 120 °C, a thermal sensitivity of 100 mK, and a thermal resolution of 160 × 120. The accuracy of the FLIR ONE camera is ±3 °C or ±5%, typical of the differences obtained with ambient temperature, and the reading resolution is 0.10C (applicable 60 s after start-up when the unit is within 15 °C to 35 °C and the scene is within 5 °C to 120 °C).

The images were taken freehand, without the use of a tripod. To maintain uniformity, images were taken at a fixed one-meter distance. The imaging procedure included images of the anterior torso (the chest and forehead) and each leg. The male participants were asked to remove their top clothing, while the female participants were requested to wear sports bras. The angle of the image was maintained at a constant value with the camera set parallel to the body of the participant (anterior view) while seated in an upright position. Each time, an image of the chest and forehead was followed by an image of the legs. Images were captured at rest, before the test (after the warm-up), immediately after the test, and every 60 s during the 15 min recovery period (Figure 1).

### 2.2. Thermal Image Processing

**Figure 2:**
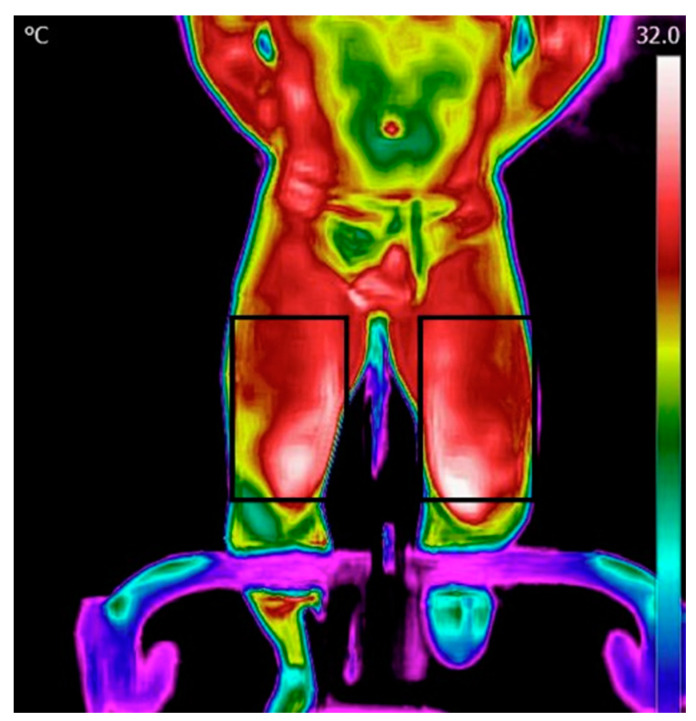
Regions of interest for the legs.

Processing and analysis of the images were performed using the FLIR Tools application. The data was then transferred into Excel and thereafter processed using MATLAB. The initial step involved reading a matrix containing temperature values within the region of interest (ROI). Thereafter, the temperature map was displayed, and an ROI was manually selected for the forehead, chest, and legs (Figure 2). Subsequently, the algorithm computed the mean and maximum temperature in each ROI.

### 2.3. Statistical Analysis

Variables are expressed as mean and standard deviation unless stated otherwise. For each ROI, we measured the change in body surface temperature values between pre- and post-exercise and every minute during the recovery period. Differences between values were calculated using repeated one-way ANOVA measurements. Paired *t*-tests were used to compare temperature changes within the same individuals (pre-test vs. post-test).

## 3. Results

Twenty-four participants (12 men and 12 women) completed the Wingate anaerobic test. Their characteristics are shown in Table 1.

**Figure 3:**
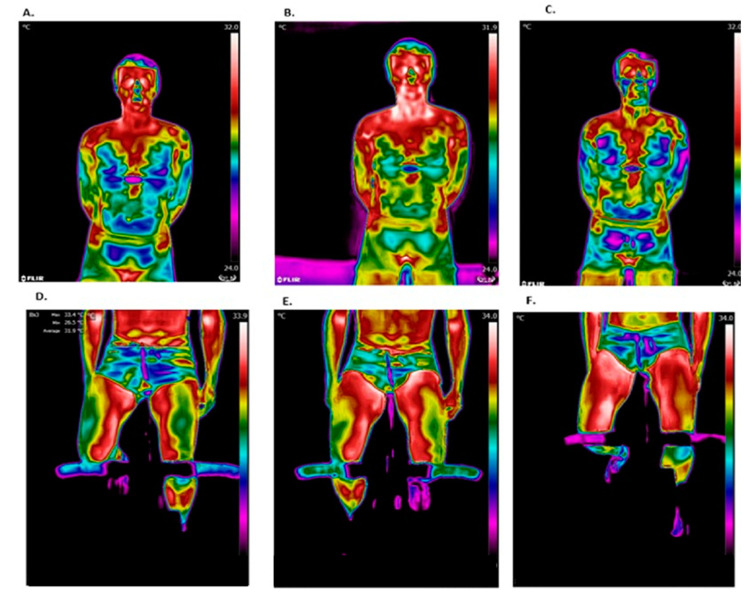
Representative thermal images. The chest region of interest (ROI): (**A**) before the test; (**B**) after the test; and (**C**) after 15 min of recovery. The leg ROIs: (**D**) before the test; (**E**) after the test; and (**F**) after 15 min of recovery.

Figure 3 shows representative thermal images during different stages of this study. Chest temperature was observed to increase after the test but decrease during recovery, while leg temperature continued to increase during the recovery period.

**Figure 4:**
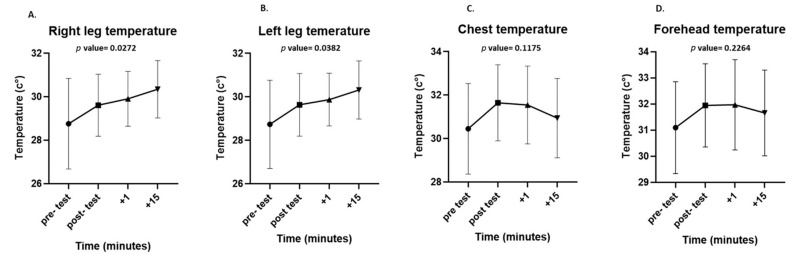
Differences in surface temperatures at all measured regions of interest (ROIs): right leg (**A**), left leg (**B**), chest (**C**), and forehead (**D**) at each time point in the experiment (pre-test, post-test, after 1 min, and after 15 min of recovery). Data are presented as mean and standard deviation.

The maximum surface temperatures of each ROI were significantly higher immediately after the test than before the test (chest: 32.43 ± 2 °C vs. 33.35 ± 1.57 °C, *p* = 0.0455; forehead: 32.74 ± 2.3 °C vs. 33.7 ± 1.43 °C, *p* = 0.0444; legs: 30.62 ± 2.1 °C vs. 31.51 ± 1.69 °C, *p* = 0.0323). During the recovery period, there was a significant increase in the surface temperature of both legs (right leg, *p* = 0.0272; left leg, *p* = 0.0382), while there was a non-significant decrease in the surface temperature of the chest (*p* = 0.1175) and forehead (*p* = 0.2264). Even after 15 min of passive recovery, the surface temperature did not return to baseline in any of the ROIs (Figure 4).

**Figure 5:**
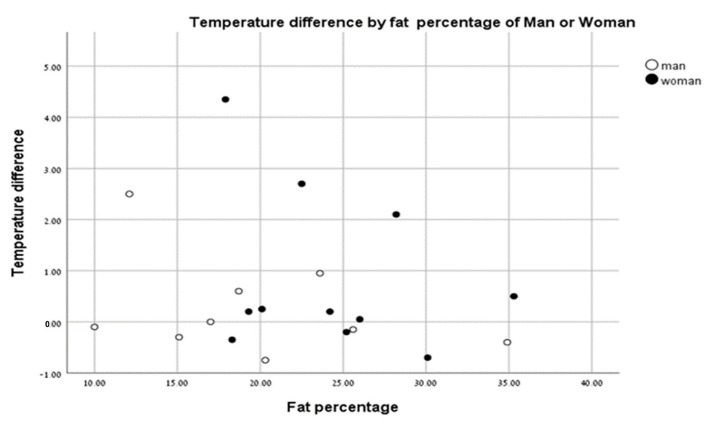
Temperature change vs. fat percentage of men (closed circles) and women (open circles).

There were no significant differences in body surface temperatures between men and women based on % body fat (Figure 5).

**Figure 6:**
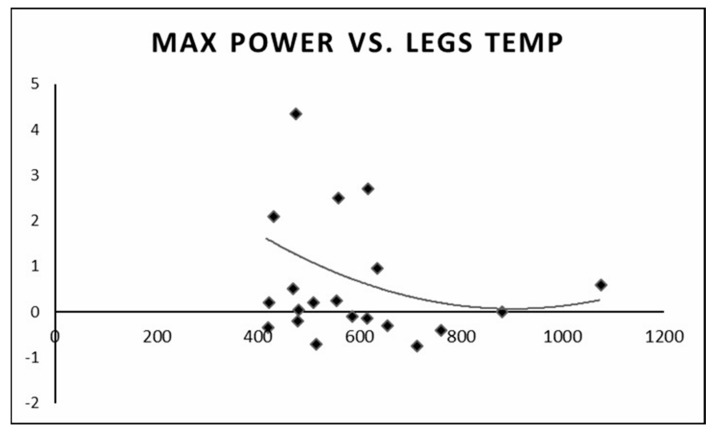
Change in leg surface temperature (pre- and post-test) versus peak anaerobic mechanical power output.

**Figure 7:**
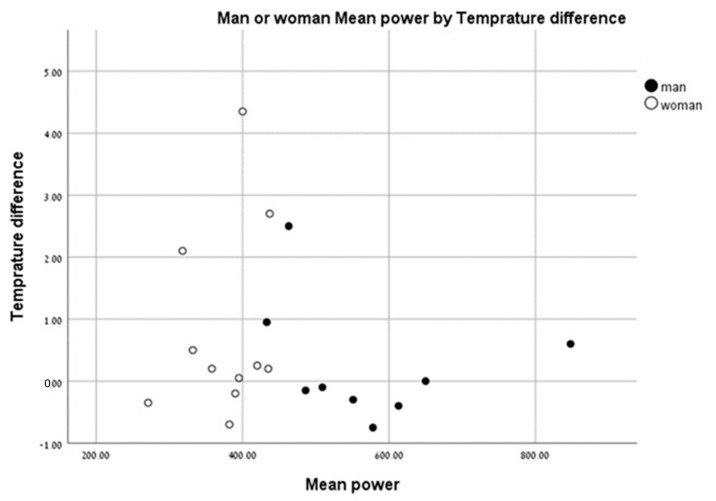
Change in leg surface temperature (pre- and post-test)) versus mean anaerobic mechanical power output.

We found that individuals with a higher anaerobic peak mechanical power output had the least change in temperature between the pre- and post-test values. Individuals with a low peak mechanical anaerobic power output showed a tendency for higher leg surface temperature change (Figure 6). The same pattern was observed for the mean anaerobic mechanical power output (Figure 7).

## 4. Discussion

This study examined body surface temperature changes during high-intensity, short-term anaerobic exercise using thermal imaging. We found that there was a significant increase in the maximum temperature in all ROIs (thigh, chest, and forehead) from pre-test to post-test values. Furthermore, we saw different thermal behaviors between the ROIs during the 15 min recovery period. The leg temperature increased significantly, whereas the chest and forehead temperatures began to decrease.

Current research that employs infrared thermography during exercise has predominantly focused on the exercise stress test, aerobic exercise training, and some resistance training [1,10,16,17,18]. To the best of our knowledge, this is the first study to examine body surface temperature changes following short-term, high-intensity, “all out” anaerobic exercise. Our study results indicated that individuals with a high anaerobic mechanical power output had the least change in body surface temperature between pre- and post-test, whereas individuals with a low anaerobic mechanical power output had a larger change in body surface temperature. Further studies are needed to understand whether this observation is an acclimatization issue or an adaptation to exercise training.

In a previous study, we tested temperature changes and distribution (entropy) of the torso associated with aerobic exercise using an incremental exercise stress test [7]. We found no significant changes in skin temperature of the limbs, chest, and forehead. This is in contrast to our results here where we observed significant changes in the skin temperature of the leg muscles, chest (representing the heart as a heat generator), and forehead (representing the heat conducted to the head/brain) with anaerobic exercise. The increase in temperature in the chest and forehead was unexpected because we hypothesized there would be an immediate decrease in temperature as blood was directed to the legs following the conclusion of the exercise [13,19]. These increases in upper body surface temperatures may reflect an increase in cardiac activity, which also affects core blood temperature.

Previous studies that examined hemodynamic behavior after exertion indicate a large increase in blood flow to the working muscles, increased metabolic heat production, and the vasodilation of superficial blood vessels, which causes a rapid increase in the temperature measured on the surface of the skin [13,19]. This is supported by previous studies that demonstrated greater blood flow and higher temperatures in active muscle areas than in other areas [12,16,20,21].

In a study by Hussain et al. (1996), which examined the hemodynamic response of the thigh after the Wingate test using Doppler ultrasound, the researchers found that increased flow to the thigh returned to normal values only after 60 min of recovery. This finding supports our results where the temperature of the thigh continued to increase during the recovery period and did not return to baseline pre-exercise values.

Borba Neves et al. [22] showed a difference between the body temperatures of men and women at rest. When analyzing the temperature differences of men and women pre- and post-test, we saw no effect of body fat (%) in males or females. Our results indicate that % body fat does not affect skin temperature following anaerobic exercise based on sex.

This study has some limitations: (1) FLIR ONE resolution may have influenced the results, and this study should be repeated with a higher resolution camera. (2) There was considerable variation in the exercise experience of our cohort. As a result, there may have been participants with more training experience and better adaptation to heat generation and regulation during and after exercise compared to others. (3) All participants in this study were both healthy and physically active, indicating that these results may not be applicable to the general population. (4) We used only thermal imaging to assess thermal changes. The inclusion of other methods, such as skin surface thermistors, would be valuable to validate our imaging findings.

## 5. Conclusions

In conclusion, this study showed that body surface temperatures increased significantly in the thighs, chest, and forehead after the Wingate test. The magnitude of the change may be related to the anaerobic mechanical output power. Additionally, the temperature of the leg surface continues to increase for at least 15 min of recovery. Our findings are in line with previous studies that showed increased blood flow to the thigh during and after short-term, high-intensity exercise.

## Figures and Tables

**Table 1:** Participant characteristics. Data are represented as mean ± standard deviation.

	Women	Men
Age (years)	29.75 ± 3.79	31.92 ± 4.31
Height (cm)	162.58 ± 4.72	176 ± 5.9
Exercise experience (years)	9.66 ± 5.23	9.41 ± 5.39
Weight (kg)	56.83 ± 4.78	71.8 ± 10.3
Fat percentage (%)	25.16 ± 6.02	18.3 ± 7.08
Fat mass (kg)	15.05 ± 4.88	14.41 ± 7.43
Muscle mass (kg)	19.23 ± 1.74	28.52 ± 3.48
Maximum heart rate (bpm)	170.36 ± 11.05	171.8 ± 10.1
Average power (watts)	368 ± 54	597 ± 123
Maximum power (watts)	485 ± 54	752 ± 157
Maximum power per body weight (watts/kg)	8 ± 1.2	10 ± 2.2

## Data Availability

Data are unavailable due to privacy or ethical restrictions.
